# MicroRNA-140-5p Mediates Renal Fibrosis Through TGF-β1/Smad Signaling Pathway by Directly Targeting TGFBR1

**DOI:** 10.3389/fphys.2020.01093

**Published:** 2020-09-04

**Authors:** Weitang Liao, Peifen Liang, Bo Liu, Zhenjian Xu, Lili Zhang, Min Feng, Ying Tang, Anping Xu

**Affiliations:** Department of Nephrology, Sun Yat-sen Memorial Hospital, Sun Yat-sen University, Guangzhou, China

**Keywords:** microRNA-140-5p, renal fibrosis, TGF-β1/smad signaling pathway, TGFBR1, microRNA

## Abstract

Renal tubulointerstitial fibrosis is usually the final outcome of various end-stage renal diseases. Recent studies have reported that microRNAs (miRNAs) play an important role in renal fibrosis. However, the biological function of microRNAs in renal fibrosis is complicated and remains unclear. In this study, our results show that miR-140-5p expression is significantly down-regulated in mice with unilateral ureteral obstruction and human proximal tubule epithelial cells (HK2) treated with TGF-β1. The knockdown of miR-140-5p upregulates the expression levels of collagen I, collagen IV, and α-SMA, decreases E-cadherin expression, and increases Smad-2/3 phosphorylation. In contrast, the overexpression of miR-140-5p decreases the expression levels of collagen I, collagen IV, and α-SMA, enhances E-cadherin expression, and inhibits the phosphorylation of Smad-2/3 in HK2 cells treated with TGF-β1. The dual-luciferase reporter assay revealed that TGFBR1 is a direct target gene of miR-140-5p. The enforced expression of miR-140-5p significantly inhibited the expression of TGFBR1 in HK2 cells. Furthermore, the knockdown of TGFBR1 has a similar effect of miR-140-5p overexpression on blocking the TGF-β1/smad signal pathway activation. In contrast, the overexpression of TGFBR1 reverses the effect of miR-140-5p inhibition on the activation of the TGF-β1/smad signal pathway. This study demonstrates that miR-140-5p regulates the TGF-β1/smad signaling pathway by suppressing the expression of TGFBR1. Therefore, miR-140-5p may have a therapeutic potential for preventing fibrotic kidney diseases through inhibiting the TGF-β1/Smad signaling pathway by directly targeting TGFBR1.

## Introduction

Renal fibrosis is a common end-stage of various kidney diseases and an independent risk factor of renal insufficiency and renal failure (Carney, 2020). The ectomesenchymal transformation of the epithelium (epithelial–mesenchymal transition, EMT) was a source of epithelial cells under the effect of various physical and chemical factors. It is through the phenotype transformation process of ectomesenchymal cells that a phenotype is obtained. It plays an important role in embryonic development, tumor metastasis, and tissue fibrosis (Carew et al., 2012; Allison, 2015). More and more evidences showed that EMT is a typical feature of renal fibrosis, and EMT of tubular epithelial cells contributes significantly to the occurrence, development, and pathological processes of kidney fibrosis (Sharma, 2014; Sun et al., 2017). Transforming growth factor-β1 (TGF-β1) is a typical pro-fibrotic cytokine involved in the process of EMT (Zhao et al., 2018). Studies had shown that TGF-β1 participates in the EMT process by activating the TGF-β1/Smad signaling pathway (Du et al., 2015). During the process of EMT, TGF-β1 and its downstream signaling molecules, such as smad2, smad3, collagen I, collagen IV, as well as α-SMA, were all increased in expression. However, the epithelial marker E-cadherin was decreased (Wang et al., 2016; Ma and Meng, 2019). MicroRNAs (miRNAs) are single-stranded non-coding small molecules. MiRNAs degraded or inhibited gene translation by fully or partially binding to the 3’-untranslated region (3’UTR) of target genes at the post-transcriptional level, thereby playing a key role in regulating protein expression (Du and Zamore, 2005; Xi et al., 2018). In recent years, more and more studies had found that miRNA is involved in the regulation of TGF-β/Smad signaling pathway and has become an important regulator of fibrosis diseases (Fan et al., 2020). Many microRNAs, such as the miR-200 family (Yang et al., 2012), miR-29 (Wang et al., 2012; He et al., 2013), miR-21 (He et al., 2014), and miR-192 (Putta et al., 2012), had been fully demonstrated for their role in the process of fibrosis. It was suggested that these microRNAs directly bind to the components of TGF-β1 to regulate the activation of signal pathways (Chung and Lan, 2015). MicroRNA-140-5p (miR-140-5p) originated from miR140-5p and was previously thought to be ubiquitous in chondrocytes during embryonic bone development (Green et al., 2015). In our previous study, we found that miR-140-5p could alleviate cisplatin-induced oxidative stress by regulating the Nrf2/ARE signaling pathway (Liao et al., 2017). Additionally, in diabetic nephropathy, the expression of miR-140-5p in kidney tissues was significantly down-regulated, and miR-140-5p protected HK-2 cells from high glucose-induced injury by inhibiting the TLR4/NF-kappaB pathway (Su et al., 2020). Several studies suggested that miR-140-5p may be linked to fibrosis in some diseases. miR140-5p was down-regulated in patients with systemic scleroderma (SSc) and miR140-5p regulates about 461 target genes, including 12 target genes that were correlated with SSc pathogenesis, which indicated that miR140-5p is a potential diagnostic biomarker and is a probable causative agent involved in the pathogenesis of skin fibrosis (Li et al., 2012). More importantly, TGFBR1 was the direct target gene of miR-140-5p. In the development of hepatocellular carcinoma, lncRNA AK002107 up-regulated the expression of TGFBR1 while inhibiting the expression of miR-140-5p to induce EMT (Tang et al., 2019). Moreover, miRNA-140-5p was involved in Wilms tumor progression by targeting the TGFBR1/SMAD2/3 and the IGF-1R/AKT signaling pathways (Liu et al., 2019). Based on the above results, we used a unilateral ureteral obstruction (UUO) mouse model and *in vitro* cell model to investigate whether miRNA-140-5p could attenuate renal fibrosis progression by regulating the TGF-β1 signaling pathway.

## Materials and Methods

### UUO Animal Model

Male C57BL/6 mice were obtained from the Animal Research Center of Sun Yat-sen University. All protocols were approved by the Sun Yat-sen University Institutional Animal Experimental Ethics Committee. A UUO kidney disease model was induced in male C57BL/6 mice (20–30 g) by ligation of the left ureter following a midline laparotomy.

### Cell Culture

HK2 cell line was purchased from the Cell Bank of Chinese Academy of Medical Sciences (Shanghai, China) and cultured in a cell incubator under 5% CO_2_ at 37°C in Dulbecco’s modified Eagle medium (Gibco, United States) supplemented with 10% fetal bovine serum (Gibco, United States).

### Cell Transfection

For cell transfection, negative control, TGFBR1 siRNA, miR-140-5p mimics, miR-140-5p inhibitor, TGFBR1-expressing vectors, and their respective negative controls (Shanghai GenePharma, China) were diluted with OptiMEM I medium at a selected optimal concentration and then transfected into HK2 cells with Lipofectamine 2000 (Invitrogen). The cells were collected 48 h after the transfection.

### Masson Trichrome Staining

Renal cortex tissues were dehydrated in a series of increasing concentrations of ethanol, embedded in paraffin after having been fixed in 4% buffered paraformaldehyde for 24 h at 4°C, and then cut in 4 mm slices. The slices were rehydrated with 100, 95, 70, and 50% alcohol and stained with Masson trichrome reagent for 15 min, followed by counterstaining with hematoxylin. According to the percentage of the area occupied by Masson trichrome-positive area, they were scored as follows: no fibrotic area scores 0, 10% of fibrotic area scores 1, 11–25% fibrotic area scores 2, 26–45% fibrotic area scores 3, 46–75% fibrotic area scores 4, and over76% of fibrotic area scores 5.

### Luciferase Assay

Using restriction enzyme site primers, a human wild-type TGFBR1 3’UTR fragment containing the conserved binding site of miR-140-5p was generated by PCR and cloned into the pMIR vector, which is referred to as wild-type TGFBR1 3’UTR. The mutant TGFBR1 3’UTR was synthesized by mutating the miR-140-5p binding site of TGFBR1 3’UTR wild-type (WT) and inserted into the equivalent reporter vector. Human 293T cells were co-transfected with 3’-UTR luciferase reporter vector containing TGFBR1 [WT or mutant (Mut)] and miR-140-5p fragment or miR-NC. At 48 h after transfection, luciferase activity was detected using the dual-luciferase reporter gene detection system (Promega Corporation).

### Quantitative Real-Time PCR

Total RNA from HK2 cells was obtained using TRIzol (Ambion) according to the manufacturer’s instructions. Complementary DNA was obtained from 1 μg of total RNA by using the special PrimeScript RT reagent kit (TaKaRa, Japan). Real-time PCR was performed using a SYBR Green qRT-PCR kit (TaKaRa, Japan) and an ABI Step One Plus Real-Time PCR System (Applied Biosystems, United States). Each test was performed in triplicate. The primers used are listed as follows:

140-5p-forward primer : FCGCATGGCAGTGGTTTTAC CCTA

140-5p-loop-RT primer : GTCGTATCCAGTGCAGGGTCCGAGG

TATTCGCACTGGATACGACCTACCA

140-5p-reverse primer: ATCCAGTGCAGGGTCCGAGG

U6-forward primer: AGAGAAGATTAGCATGGCCCCTG

U6-loop-RT primer : GTCGTATCCAGTGCAGGGTCCGAG GTATTCG

CACTGGATACGACAAAATA

U6-reverse primer: ATCCAGTGCAGGGTCCGAGG

TGFBR1-F: AAACTTGCTCTGTCCACGG

TGFBR1-R: AATGGCTGGCTTTCCTTG

α-SMA-F: CTGACAGAGGCACCACTGAA

α-SMA-R: CATCTCCAGAGTCCAGCACA

Col-I-F: GATTGAGAACATCCGCAGC

Col-I-R: CATCTTGAGGTCACGGCAT

Col-IV-F: ATCTCTGCCAGGACCAAGTG

Col-IV-R: CGGGCTGACATTCCACAAT

The relative mRNA expression changes were calculated by using the 2^–Δ*Ct*^ method.

### Western Blot Analysis

Renal tissue and HK2 cells were lysed with radioimmunoprecipitation assay lysis buffer and phenylmethylsulfonyl fluoride (Beyotime, China). The bicinchoninic acid protein detection kit (Boster, China) is used to detect the protein concentration in the sample. First, total protein samples were separated by sodium dodecyl sulfate–polyacrylamide gel electrophoresis. They are then printed on the Millipore (United States) film. After blocking non-specific protein binding, the Western blot was incubated with a primary antibody at 4°C overnight. The dilution ratio of primary antibodies was: 1:3,000 for β-actin (Sigma), 1:3,000 for TGF-β1 (Sigma), 1:1,000 for COL-I (Sigma), 1:1,000 for COL-IV (Abclonal), 1:1,000 for α-SMA (Abclonal), E-cadherin (1:1,000), TGFBR1 (1:500), p-Smad-2/3 (1:500), and Smad-2/3 (1:1,000) (Cell Signaling Technology, MA, United States). After three phosphate-buffered saline with Tween 20 washings, the membrane was treated with HRP-labeled secondary antibodies (cell signaling technology) for 2 h. The immune complexes were detected with enhanced chemiluminescence agents. Image J (NIH, United States) software was used to quantify protein expression according to gray value.

### Statistical Analysis

SPSS software was used for statistical analysis. The data were expressed as mean ± SD, and each experiment was repeated at least three times. The statistical significance of the difference was measured by Student’s *T-*test or non-parametric test. Unless otherwise stated, *P* < 0.05 is considered as statistically significant.

## Results

### Activation of TGF-β1 Signaling Pathway and Histological Changes in UUO Mice

**FIGURE 1:**
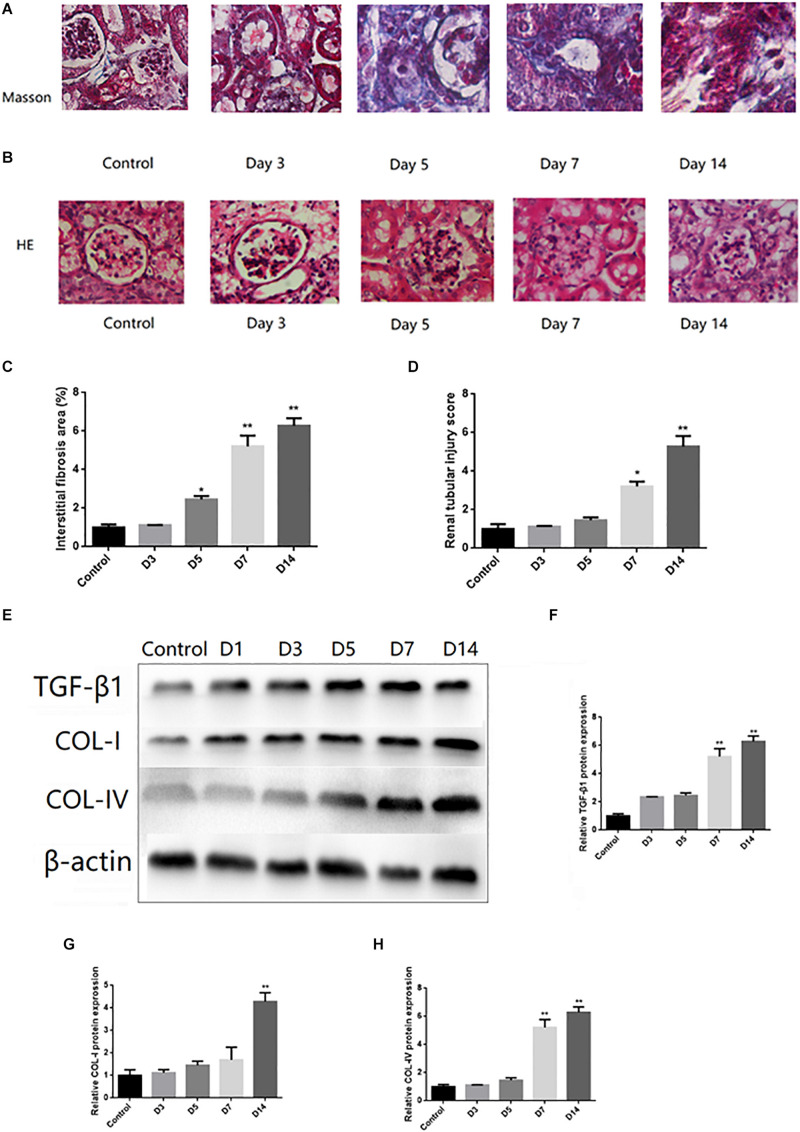
Histological changes and activation of TGF-β1 signaling pathway in UUO mice. **(A)** Representative images of Masson trichrome staining of kidney of UUO (Masson trichrome, ×400). **(B)** Representative images of renal tubular injury of kidney of UUO (H&E, ×400). **(C)** Quantitative analysis of tubular interstitial fibrosis from Masson trichrome staining results. **(D)** Quantitative analysis of tubular injury from HE staining results. **(E–H)** The protein expression levels of TGF-β1, COL-I, and COL-IV were tested by Western blot analysis. ^∗^*P* < 0.05 versus Control; ^∗∗^*P* < 0.01 versus Control.

The pathological changes of the kidney were examined by Masson trichromatic staining. Compared to the control group, increased deposits of extracellular matrix (ECM) in the renal tubulointerstitium and fibrotic areas were shown in UUO mice (Figures 1A,C). In addition, H&E staining showed mesangial cell proliferation, ECM increase, glomerular basement membrane thickening, and a disordered arrangement of renal tubular cells in UUO mice (Figures 1B,D). The Western blots showed that TGF-β1, COL-I, and Col-IV were increased in mice after UUO surgery (on 3, 7, and 14 days, respectively) in a time-dependent tendency (Figures 1E–H).

### Expression of miR-140-5p in UUO Mice and HK2 Cells

**FIGURE 2:**
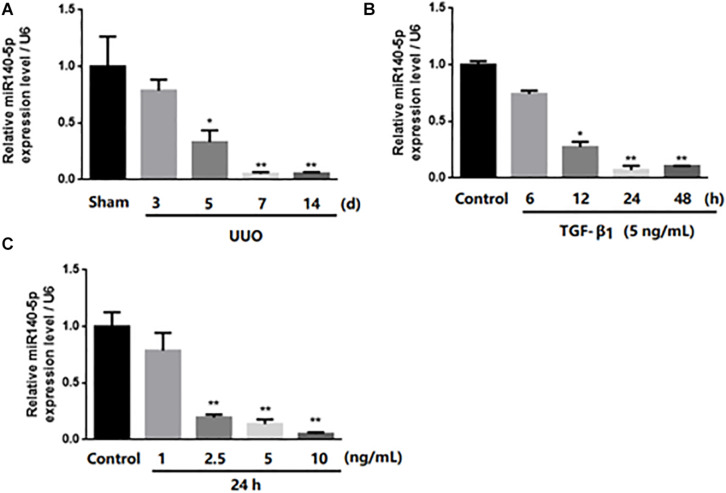
Expression of miR-140-5p in UUO mice kidney and level of miR-140-5p in HK2 cells after TGF-β1 treatment. **(A)** miR-140-5p expression of UUO mice kidney was determined by RT-qPCR at indicated time points after UUO treatment. **(B)** Time-dependent decrease of miR140-5p expression. Expression of miR-140-5p in HK2 cells exposed to 5 ng/ml TGF-β1 treatment for 6, 12, 24, 48 h. miR140-5p level was determined by RT-qPCR. **(C)** Dose-dependent decrease of miR140-5p expression. HK2 cells were treated with different concentrations of TGF-β1 for 24 h Data was presented as mean ± SD of three experiments. Control: no transfection treatment, Negative control (NC): cells transfected with scrambled oligonucleotide. ^∗^*P* < 0.05 versus Control; ^∗∗^*P* < 0.01 versus Control.

In order to study the potential role of miR-140-5p in renal fibrosis, we analyzed the expression changes of miR-140-5p in UUO mice. As shown in Figure 2A, the expression of miR-140-5p in mice from the UUO group was significantly reduced at 3, 5, 7, and 14 days in a time-dependent manner after the UUO operation. To further explore the potential role of miR-140-5p in renal fibrosis, TGF-β1 was used to stimulate HK-2 cells, and the expression of miR-140-5p was detected by qRT-PCR. As shown in Figure 2B, we found that the expression of miR-140-5p was significantly decreased at different time points (6, 12, 24, and 48 h). Then, after treating the HK2 cells with different concentrations of TGF-β1, we measured the expression of miR-140-5p. We found that the level of miR-140-5p is significantly decreased in a dose-dependent manner (Figure 2C). These results suggested that the down-regulation of miR-140-5p levels in UUO mice and HK2 cells after TGF-β1 treatment may be related to renal fibrosis.

### Effect of miR-140-5p on TGF-β1/Smad Signaling Activation

**FIGURE 3:**
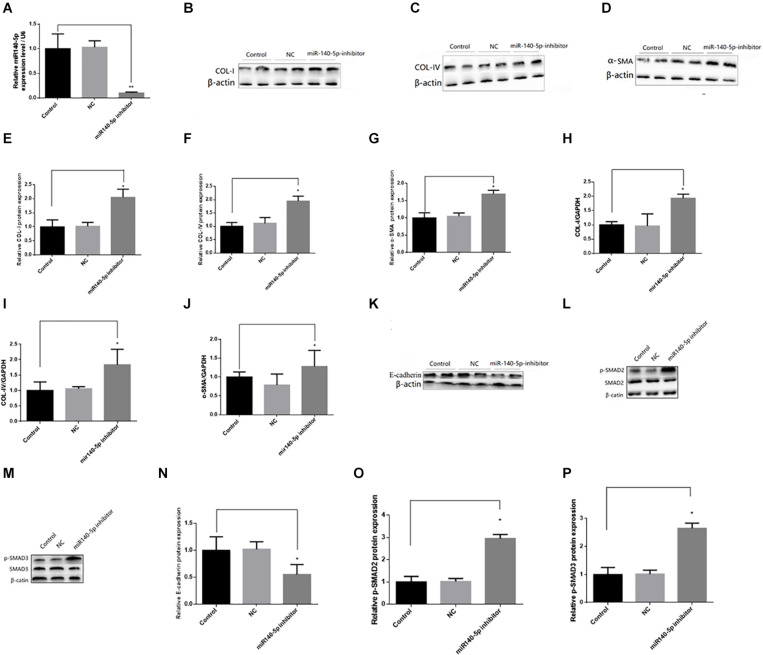
Knockdown of miR140-5p aggravated TGF-β1-induced fibrosis and EMT in HK2 cells. HK2 cells were transfected with miR140-5p inhibitor (20 nmol/L) or inhibitor negative control (NC, 20 nmol/L) for 24 h and then treated with TGF-β1 (5 ng/ml) for 48 h. **(A)** the expression level of miR140-5p in HK2 cells transfected with miR140-5p inhibitor. The protein expression of COL-I **(B)**, COL-IV **(C),** α-SMA **(D)** were examined by Western blotting in HK2 cells. **(E–G)** The bar chart showed the ratio of quantitative band density measurements of COL-I, COL-IV, and α-SMA to β-actin, respectively. The mRNA expression of COL-I **(H)**, COL-IV **(I)**, and α-SMA **(J)** were examined by RT-qPCR in HK2 cells. The protein expression of E-cadherin **(K)**, p-SMAD2/SMAD2 **(L)**, p-SMAD3/SMAD3 **(M)** were examined by Western blotting in HK2 cells. β-actin was detected as a loading control. **(N–P)** The bar chart showed the ratio of quantitative band density measurements of E-cadherin, p-SMAD2, p-SMAD3 to β-actin, respectively. Data was presented as mean ± SD of three experiments. Control: no transfection treatment, Negative control (NC): cells transfected with scrambled oligonucleotide. ^∗^*P* < 0.05 versus Control; ^∗∗^*P* < 0.01 versus Control.

**FIGURE 4:**
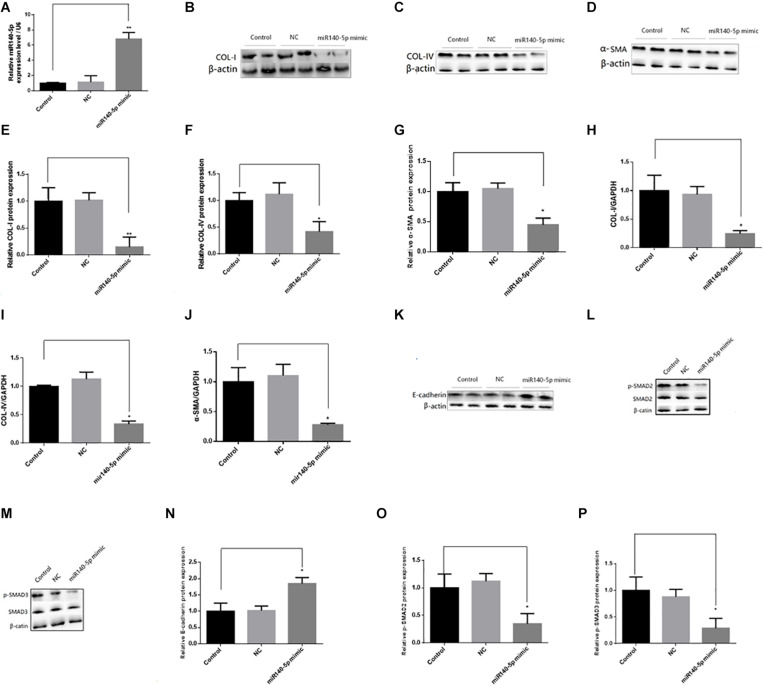
Overexpression of miR140-5p inhibited TGF-β1-induced fibrosis and EMT in HK2 cells. HK2 cells were transfected with miR140-5p mimic (20 nmol/L) or mimic negative control (NC, 20 nmol/L) for 24 h and then treated with TGF-β1 (5 ng/ml) for 48 h. **(A)** the expression level of miR140-5p in HK2 cells transfected with miR140-5p mimic. The protein expression of COL-I **(B)**, COL-IV **(C)**, α-SMA **(D)** were examined by Western blotting in HK2 cells. **(E–G)** The bar chart showed the ratio of quantitative band density measurements of COL-I, COL-IV, and α-SMA to β-actin, respectively. The mRNA expression of COL-I **(H)**, COL-IV **(I)**, and α-SMA **(J)** were examined by RT-qPCR in HK2 cells. The protein expression of E-cadherin **(K)**, p-SMAD2/SMAD2 **(L)**, p-SMAD3/SMAD3 **(M)** were examined by Western blotting in HK2 cells. β-actin was detected as a loading control. **(N–P)** The bar chart showed the ratio of quantitative band density measurements of E-cadherin, p-SMAD2, p-SMAD3 to β-actin, respectively. Data was presented as mean ± SD of three experiments. Control: no transfection treatment, Negative control (NC): cells transfected with scrambled oligonucleotide. ^∗^*P* < 0.05 versus Control; ^∗∗^*P* < 0.01 versus Control.

To investigate whether miR-140-5p could affect TGF-β1/smad signaling pathway activation, we first transfected the HK2 cells with the miR-140-5p inhibitor, which significantly reduced the expression level of miR-140-5p (Figure 3A). Then, these were treated with TGF-β1 (5 ng/ml) for 24 h. We found that the inhibition of miR-140-5p significantly increased the TGF-β1-induced expression of collagen I, collagen IV, and α-SMA in both protein and RNA levels (Figures 3B–J). E-cadherin is a specific biomarker of renal EMT. As shown in Figures 3K,N, when miR-140-5p was knocked down, the protein level of E-cadherin was also down-regulated. SMAD2 and SMAD3 are important receptors in the early stage of the TGF-β signaling pathway. We knocked down miR-140-5p to investigate whether the phosphorylation of SMAD2 and SMAD3 in HK2 cells was affected. As shown in Figures 3L,M,O,P, the down-regulation of miR-140-5p significantly increased the levels of phosphorylated SMAD2 and SMAD3 in HK2 cells. Next, we transfected miR-140-5p mimics into HK2 cells, which significantly increased the expression level of miR-140-5p (Figure 4A), and then these were treated with TGF-β1 (5 ng/ml) for 24 h to investigate the effect of miR-140-5p overexpression on the activation of the TGF-β1/smad signaling pathway. With the up-regulation of miR-140-5p, the protein and the RNA levels of collagen I, collagen IV, and α-SMA decreased significantly (Figures 4B–J). However, the expression of cadherin increased significantly (Figures 4K,N). Moreover, the phosphorylation of Smad-2/3 (Figures 4L,M,O,P) decreased significantly. These findings demonstrated that miR-140-5p may play an important role in TGF-β1-mediated renal fibrosis, thereby protecting HK2 cells from TGF-β1-induced fibrosis.

### miR-140-5p Directly Acted on the 3’-UTRs of TGFBR1

**FIGURE 5:**
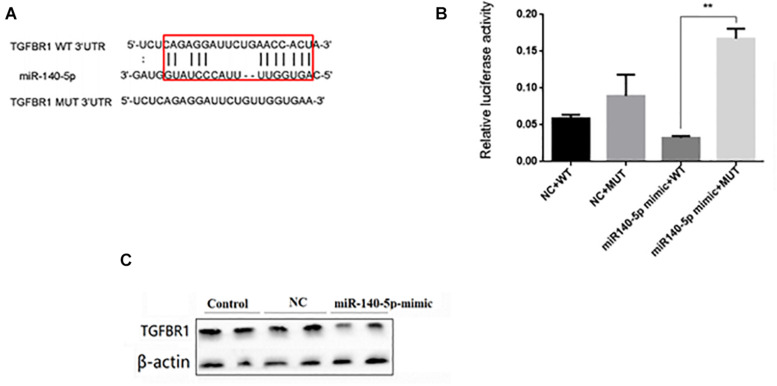
miR140-5p negatively regulated TGFBR1 expression. **(A)** Bioinformatics analysis of predicted interactions of miR-140-5p with its binding sites in TGFBR1 3’UTR. **(B)** Luciferase activity was measured by dual-luciferase reporter assay. Luciferase activity was normalized to Renilla luciferase activity. Overexpression of miR-140-5p significantly increased luciferase activity in cells transfected with TGFBR1 3’ UTR-WT, but not TGFBR1 3’ UTR-NC or TGFBR1 3’ UTR-mut. **(C)** HK2 cells transfected with miR-140-5p or NC were harvested and TGFBR1 protein level was analyzed by western blot. β-actin was detected as a loading control. Data was presented as mean ± SD of three experiments. Control: no transfection treatment, Negative control (NC): cells transfected with scrambled oligonucleotide. ^∗∗^*P* < 0.01 versus Control.

In order to reveal the molecular mechanism of miR-140-5p affecting renal fibrosis, we used TargetScan and MiRbase to analyze its predicted target genes. Among the predicted targets, we found that TGFBR1 may be a functional target gene of miR-140-5p for renal fibrosis because it plays a key role in mediating the TGF-β1 signal pathway (Figure 5A). We then performed luciferase reporter analysis. The dual-luciferase reporter assay showed that miR-140-5p inhibited the fluorescence activity of the wild-type reporter plasmid of TGFBR1 3’UTR but had no similar inhibitory effect on the mutants, thus further confirming the targeted regulatory effect of miR-140-5p on TGFBR1 (Figure 5B). We then assessed whether the overexpression of miR-140-5p would affect the expression level of TGFBR1. We then transfected HK2 cells with miR-140-5p mimics or control oligonucleotides. Consistent with the dual-luciferase reporter assay, the upregulation of miR-140-5p significantly reduced the TGFBR1 protein level. We then evaluated whether the overexpression of miR-140-5p would affect the expression of TGFBR1. In this experiment, we transfected the HK2 cells with miR-140-5p mimic or control oligonucleotides. Consistent with the changes in TGFBR1 3’UTR reporter luciferase, the upregulation of miR-140-5p significantly decreased the levels of the TGFBR1 protein at 24 h post-transfection (Figure 5C). In summary, these results confirmed that TGFBR1 is the target gene of miR-140-5p and that miR-140-5p can directly target TGFBR1 3’UTR.

### Downregulation of TGFBR1 Inhibited the Activation of the TGF-β/Smad Signaling Pathway

**FIGURE 6:**
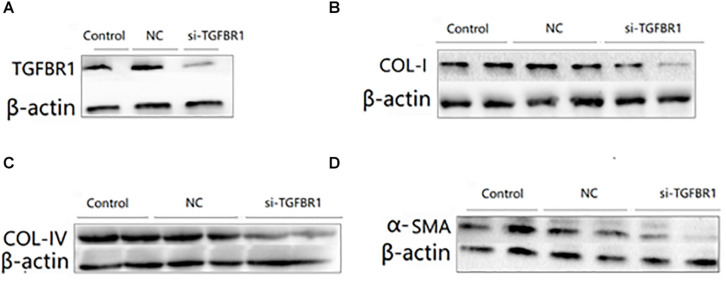
The effects of TGFBR1 knockdown on TGF-β1-induced collagen accumulation and cell differentiation in HK2 cells. HK2 cells were transfected with si-TGFBR1 or NC, and then treated with TGF-β1 (5 ng/ml) for 24 h. The knock down effects of si-TGFBR1 was confirmed by Western blot **(A)**. The protein expression of COL-I **(B)**, COL-IV **(C)**, and α-SMA **(D)** was determined by Western blot. β-actin was detected as a loading control. Data was presented as mean ± SD of three experiments. Control: no transfection treatment, Negative control (NC): cells transfected with scrambled oligonucleotide.

In order to study the functional role of TGFBR1, we first transfected the HK2 cells with si-TGFBR1 and then treated these with TGF-β1 (5 ng/ml) for 24 h. The Western blot analysis showed that the protein level of TGFBR1 was significantly reduced in HK2 cells transfected with si-TGFBR1 (Figure 6A). With the downregulation of TGFBR1, the expression of collagen I, collagen IV, and α-SMA dramatically decreased in HK2 cells transfected with TGF-β1 (Figures 6B–D). These results indicated that the overexpression of miR-140-5p may protect the HK2 cells from TGF-β1-induced renal fibrosis by inhibiting TGFBR1 expression.

### Overexpression of TGFBR1 Reversed miR-140-5p-Reduced TGF-β1 Activation in HK2 Cells

**FIGURE 7:**
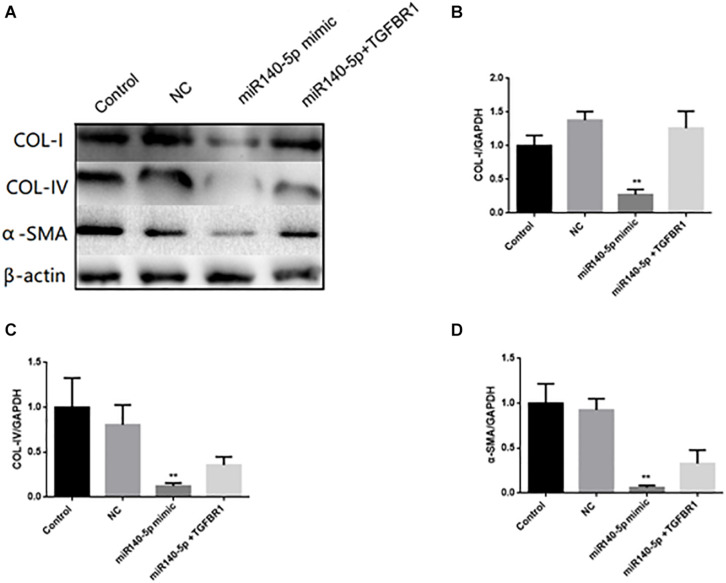
TGFBR1 was involved in the effects of miR-140-5p on TGF-β1-induced collagen accumulation and cell differentiation in HK2 cells. HK2 cells were transfected with miR-140-5p mimic and TGFBR1-expressing vectors, and then treated with TGF-β1 (5 ng/ml) for 24 h. **(A)** The protein expression of COL-I, COL-IV and α-SMA was determined by Western blot. β-actin was detected as a loading control. (**B–D)** The mRNA level of COL-I, COL-IV, and α-SMA was determined by qRT-PCR. Data was presented as mean ± SD of three experiments. Control: no transfection treatment, Negative control (NC): cells transfected with scrambled oligonucleotide. ^∗∗^*P* < 0.01 versus Control.

To assess whether TGFBR1 was a key functional target of miR-140-5p in HK2 cells, we co-transfected the HK2 cells with miR-140-5p mimic and TGFBR1-expressing vectors and then treated these with TGF-β1 (5 ng/ml) for 24 h. We found that the overexpression of miR-140-5p significantly inhibited the protein and the mRNA levels of COL I, COL IV, and α-SMA in TGF-β1-treated HK-2 cells. However, with the miR-140-5p and TGFBR1 up-regulated at the same time, we found that these above-mentioned effects were significantly reversed (Figure 7). Therefore, the inhibition of TGF-β1 activation by miR-140-5p was reversed in the cells co-expressing TGFBR1. In summary, these results suggested that miR-140-5p may inhibit renal fibrosis by targeting the TGFBR1-mediated TGF-β1/smad signaling pathway.

## Discussion

Renal fibrosis was an inevitable outcome of many chronic kidney diseases and eventually leads to end-stage renal failure (Xiao and Liu, 2013). It was widely believed that, among various pathogenic factors, TGF-β1 and its downstream Smad signaling pathway play an important role in renal fibrosis (Xi et al., 2018).

In recent years, more and more studies had reported that miRNA is involved in the occurrence and the development of renal fibrosis, and TGF-β1 plays an important role in renal fibrosis by regulating the expression of miRNAs (Qin et al., 2011; Zhong et al., 2011; Wang et al., 2014; Meng et al., 2016). However, there had been no report on whether miR-140-5p participates in the process of renal fibrosis. To explore the functional role of miR-140-5p in the pathological processes of renal fibrosis, we established a UUO mouse model. Our results showed that miR-140-5p is significantly decreased in the kidney cortex during UUO-induced renal fibrosis (Figure 2A). To further determine whether the decrease of miR140-5p expression was involved in renal fibrosis, we then analyzed the expression of miR140-5p in HK-2 cells stimulated with TGF-β1. As shown in Figures 2B,C, miR140-5p expression was significantly reduced in a time- and dose-dependent manner. These results concurred with a report that miR-140-5p is decreased in fibrotic skin tissues of patients with systemic scleroderma, suggesting that miR140-5p may be involved in the process of fibrosis (Li et al., 2012). The TGF-β1/Smad signal pathway was considered to be the primary pathway for fiber formation. Smad2 and Smad3 proteins were phosphorylated when the TGF-β receptor was activated (Loboda et al., 2016). To investigate the possible involvement of miR-140-5p in this process, HK2 cells were transfected with miR-140-5p inhibitors and then treated with TGF-β1. Our results indicated that the knockdown of miR-140-5p increases the level of phosphorylation of SMAD2 as well as that of SMAD3 (Figures 3L,M). However, when HK2 cells were transfected with miR-140-5p mimics and then treated with TGF-β1, we found that the phosphorylation level of SMAD2/3 both decreased (Figures 4L,M).

ECM was characterized by increased collagen (e.g., types I and IV) and down-regulation of epithelial markers (e.g., E-cadherin) (Che et al., 2017). Furthermore, the formation of myoblast cells characterized by high-SMA expression was one of the key events in renal fibrosis (Ina et al., 2011). We found that silencing miR-140-5p significantly increased the TGF-β1-induced expression of type I collagen, type IV collagen, and celloma-SMA, while it reduced the expression of E-cadherin in HK2 cells (Figures 3B–K). In contrast, the overexpression of miR-140-5p inhibited the TGF-β1-induced synthesis of type I collagen, type IV collagen, and α-SMA, while it induced the expression of E-cadherin in HK2 cells (Figures 4B–K). Overall, these findings strongly suggest the functional role of miR-140-5p in TGF-β1/smad-mediated renal fibrosis and indicate the upregulation of miR-140-5p reversed TGF-β1-induced HK2 cell differentiation and collagen generation, leading to the protection of HK2 cells from TGF-β1-induced renal fibrosis.

In order to explore the mechanism of miR-140-5p in the pathogenesis of renal fibrosis, we identified that TGFBR1 is a direct target of miR-140-5p in humans and mice. TGFBR1 was the key component involved in the TGF-β/Smad signaling pathway. However, the role of TGFBR1 in renal fibrosis was still poorly studied. A previous study had found that knockdown TGFBR1 inhibits TGF-β signaling pathway activation, thereby blocking the differentiation of cardiac fibrosis (Cheng et al., 2017). Yang et al. (2019) found that progranulin promotes skin sclerosis by up-regulating TGFBR1 and enhancing the TGF-β/Smad3 signaling pathways. Zhao et al. (2015) reported that the overexpression of miR-101a inhibited hypoxia-induced myocardial fibrosis by down-regulating TGFBR1 on cardiac fibrosis. However, the role of miR-140-5p in TGF-β1-induced renal fibrosis through targeting TGFBR1 had not been reported. Luciferase reporter plasmids containing predictive TGFBR1 target gene sequences showed that TGFBR1 is a direct target of miR-140-5p (Figure 5B). Consistent with miR-140-5p targeting the mRNA of TGFBR1, we found that the miR-140-5p mimic could reduce the expression of TGFBR1 in HK2 cells (Figure 5C), suggesting that TGFBR1 is negatively regulated by miR-140-5p. In addition, the inhibition of TGFBR1 and the overexpression of miR-140-5p had a similar renal protective effect (Figure 6), while the transfection of the TGFBR1-expressing vector partially blocked the protective effect of miR-140-5p overexpression (Figure 7). Overall, our results suggested that miR-140-5p may protect the HK2 cells from TGF-β-induced renal fibrosis by directly targeting TGFBR1. Therefore, upregulating the expression of miR-140-5p to block the activation of the TGF-β/Smad signaling pathway may be a promising method for the treatment of renal fibrosis.

## Data Availability Statement

All datasets presented in this study are included in the article/supplementary material.

## Ethics Statement

The animal study was reviewed and approved by the Sun Yat-sen University institutional animal care and use committee.

## Author Contributions

WL contributed to the conceptualization, methodology, data curation, and writing of the original draft. PL contributed to the methodology, resources, and data curation. BL contributed to the methodology, software, and data curation. ZX contributed to the methodology and the data curation. LZ contributed to the investigation, methodology, and data curation. MF contributed to the software and the data curation. YT contributed to the resources and data curation. AX contributed to the supervision and writing – reviewing and editing. All authors contributed to the article and approved the submitted version.

## Conflict of Interest

The authors declare that the research was conducted in the absence of any commercial or financial relationships that could be construed as a potential conflict of interest.
